# Spanish validation of the short version of the racing and crowded thoughts questionnaire (RCTQ-13)

**DOI:** 10.1186/s12888-024-05618-1

**Published:** 2024-03-19

**Authors:** Daniela Garcés Rodríguez, Juan Pablo Zapata-Ospina, María Mercedes Uribe, Diana Suarez, Luis Fernando Tabares, Luisa Fernanda Ahunca, Daniel Camilo Aguirre, Jorge Carlos Holguín, Jenny García Valencia

**Affiliations:** 1 Department of Psychiatry, University of Antioquia, Medellin, Colombia; 2 Institute of Medical Research, University of Antioquia, Medellin, Colombia; 3 Hospital Mental de Antioquia, Bello, Colombia; 4 Hospital Alma Máter de Antioquia, Medellin, Colombia; 5 Hospital San Vicente Fundacion, Medellin, Colombia; 6 Institute of Medical Research, University of Antioquia, Medellin, Colombia; 7 Department of Psychiatry, University of Antioquia, Medellin, Colombia; 8 Department of Psychiatry, University of Antioquia, Medellin, Colombia

**Keywords:** Racing and crowded thoughts, Thinking, Psychopathology, Bipolar disorder, Depressive disorder, Anxiety, Psychometrics

## Abstract

**Background:**

The *Racing and Crowded Thoughts Questionnaire* (RCTQ-13) is the most widely used specific scale for the measurement of racing thoughts, but there is currently no Spanish version that allow the evaluation in Spanish-speaking patients. The objective of this study is to translate, adapt, and validate the RCTQ-13 in a Colombian population with affective disorders.

**Methods:**

The questionnaire was translated and back-translated, and corrections were implemented following a pilot test to improve comprehensibility. We included patients with Bipolar I Disorder and with Major depressive disorder seen in three centers in the city of Medellín, Colombia. We evaluate structural validity with confirmatory factor analysis, internal consistency, and test-retest reliability. Construct validity was also assessed with the comparison between euthymic, maniac, and depressive episodes and the correlation with worry, rumination, and mania scales. Responsiveness was measured 1 month after the first evaluation. Based on item response theory (IRT), we also estimated item difficulty, discrimination, and fit using a generalized partial credit model.

**Results:**

Two hundred fifty subjects were included. Confirmatory factor analysis revealed that the three-factor structure of the scale was appropriate. Internal consistency was adequate for the entire scale (Cronbach’s alpha = 0.95, 95% CI: 0.94-0.96) and for each factor. Test-retest reliability was good (intraclass correlation coefficient = 0.82, 95%IC: 0.70-0.88). For construct validity, we observed differences between patients with different types of affective episodes, a moderate positive correlation with the Penn State Worry Scale (r = 0.55) and the Ruminative Response Scale (r = 0.42), and a low negative correlation with the Young Mania Rating Scale (r = − 0.10). Responsiveness was proved to be adequate. Under IRT, the response thresholds for the response options are organized for all items. The infit was adequate for all items and the outfit was acceptable.

**Conclusions:**

The Spanish version of the RCTQ-13 is a reliable, valid, and responsive scale and can be used for the clinical assessment of the construct of racing and crowded thoughts in patients with the spectrum of affective disorders in whom this experience can be expressed with different nuances. Further research is needed to expand the relationship with rumination and worry.

**Supplementary Information:**

The online version contains supplementary material available at 10.1186/s12888-024-05618-1.

## Background

Racing thoughts refer to the subjective acceleration and overproduction of thoughts, which have classically been associated with mania and hypomania in bipolar disorder (BD) [1, 2]. As a concept, racing thoughts encompass different psychopathological experiences also present in mixed, depressive, and anxious states, but different terms can be recognized in the literature. “*Crowded thoughts”* is commonly described by the patient as feeling that their head is full of thoughts they cannot stop [3]; *“racing thoughts”* indicate an increased velocity of thoughts; and *“depressive ruminations”* are thoughts and ideas confined to specific situations in the past [4, 5]. Distinguishing between the different domains of racing thoughts holds great clinical and psychopathological value. Its presence or absence and the degrees it may present can guide the differential diagnosis when clinical manifestations overlap, especially in disorders characterized by affective disturbances [6]. It also allows for a deeper psychopathological understanding of the patients’ experience [7].

However, until now, efforts to diagnose these types of disturbances have focused on data obtained from the medical records. Tools for measuring this symptom are scarce, mainly due to the difficulty inherent to the subjective nature of thinking. Nonetheless, these tools are necessary, as spontaneous patient reporting is rare, and it is up to the clinicians to specifically investigate this aspect [8]. Another difficulty arises from possible fluctuations, so it may not be easy to capture the full picture. Furthermore, thought processes are not considered primary targets in the acute phases of mood disorders, where the priority for clinicians is to stabilize a patient’s clinical condition. In fact, when the scales used for depressive and anxious disorders include the velocity of thoughts, they only include items on slowness and not on acceleration. In addition, the scales used for mania and hypomania include items on racing thoughts, but do not explore their different domains [9].

For this reason, Weiner et al [8] developed the *Racing and Crowded Thoughts Questionnaire* (RCTQ) in 2018, consisting of 34 items in English that measure the number, velocity, and types of thoughts. Their conceptual framework proposes that “racing thoughts” is a multifaceted phenomenon involving three domains: 1) “thought overactivation,” referring to an excessive number and velocity of thoughts; 2) “burden of thought overactivation,” which evokes the overwhelming impact of thought overactivation; and 3) “thought overexcitability,” which describes distractibility, a distinctive characteristic associated with racing thoughts. A factor analysis was conducted on the initial validation on the BD population, and a three-factor structure was confirmed. However, it yielded redundant items, which were eliminated, giving rise to a 13-item version of the scale [10].

This short version of the RCTQ (RCTQ-13) preserves the initial three-factor structure and has been shown to have adequate internal consistency and adequate convergent, divergent, and discriminant validity. It was validated on hypomanic and mixed states, as well as on depression with subclinical hypomanic/activation symptoms. This suggests that it could be particularly sensitive to activation symptoms in BD and could become a valuable tool in providing follow-up for these patients. It could be useful in depressive and anxious disorders as well, where patients have also reported this experience [10, 11]. For this reason, it is the most widely used specific scale for this mental phenomenon, but there is currently no Spanish validation of the RCTQ-13 that would allow evaluation in Spanish-speaking patients and an item-level analysis has also not been performed. Therefore, the aim of this study was to translate, adapt, and validate the RCTQ-13 into Spanish in a sample of Colombian patients with mood disorders using classical test theory and item response theory (IRT).

## Methods

This was a multicenter study conducted in three centers in the city of Medellín, Colombia (Hospital San Vicente Fundación, Hospital Mental de Antioquia, and Hospital Alma Máter de Antioquia). It complies with the Declaration of Helsinki and was approved by the Bioethics Committee of the School of Medicine of Universidad de Antioquia (Approval Act 016 of 2021) and by the participating institutions. All participants signed the informed consent form. The first stage consisted of translation, adaptation, and the pilot test, and the second stage was for the evaluation of psychometric properties.

### Translation and adaptation

We obtained permission from the lead author (Dr. Luisa Weiner) to use the scale. The objective of this stage was to produce a Spanish version of the RCTQ-13 that would be linguistically and culturally equivalent to the original English version. The scale was translated and adapted following the translation and back-translation process. Initially, two translators independently translated the items from English to Spanish. The two translators and a review board consisting of three psychiatrists, a psychiatry resident, and a professor from the School of Languages reviewed both translations and agreed on a unified Spanish version of the instrument. This version was then translated back into English by two different translators who were not familiar with the original version of the scale. The review board and the translators compared both back-translations and resolved the inconsistencies to produce a single back-translated scale, which was then compared with the original. The found inconsistencies were modified until all board members agreed that the original and translated versions had identical meaning and content, only with the particularities specific to the Colombian population.

### Pilot test

It was conducted on 14 subjects diagnosed with BD. The aim was determining the ease of administration of the questionnaire, the average time of administration, and the difficulties that could arise during answering. Afterwards, they were subjected to a cognitive interview as recommended by the International Society for Pharmacoeconomics and Outcomes Research (ISPOR) [12] to evaluate the comprehensibility, comprehensiveness, and relevance of the items, instructions, and response options. These interviews were recorded and transcribed verbatim for later analysis by the research team.

### Validation stage

#### Participants

We included patients diagnosed with bipolar I disorder, in maniac or depressive episode or in full remission (considered euthymic), and patients with major depressive disorder following the criteria of the Diagnostic and Statistical Manual of Mental Disorders, Fifth Edition (DSM-5) [13], who were under inpatient and outpatient care in the participating institutions. Patients with cognitive impairment, intellectual disability, psychosis, and a level of education under 5 years were excluded. We calculated the sample size for each of the evaluated psychometric properties. We considered 250 people for internal consistency following Streiner’s recommendations [14] for scales with over 10 items, with an expected Cronbach’s alpha of 0.7 and a 95% confidence interval (95% CI) width of 0.1. We used the same number of patients for structural validity. We included 100 participants for the test-retest reliability, as recommended by De Vet [15] with an expected intraclass correlation coefficient (ICC) of 0.7 and a 95% CI width of 0.1. For convergent construct validity, we calculated a sample size of 55 people using the sample size formula for determining the correlation coefficient, with a type I error of 0.05, a type II error of 0.20, an alternative hypothesis correlation coefficient of 0.5 (as moderate correlations were expected with other related but not identical constructs), and a null hypothesis correlation coefficient of 0.2, as well as a one-tailed hypothesis test. For discriminant construct validity we included 63 patients in each group, calculating a sample size for the mean difference between independent groups, with a type I error of 0.05, a type II error of 0.2, an expected standardized mean difference of 0.5 and a 1:1 ratio of affected vs. unaffected [16]. For responsiveness, a sample size of 72 was calculated using the Hanley and McNeil formula [17] with an expected area under the ROC curve (AUROC) of 0.7, a type I error of 0.05, a type II error of 0.20, a null hypothesis AUROC of 0.5 and an expected 2:1 ratio between subjects who do not change and those who do.

#### Procedures

Each subject received information about the study, and they were asked to complete the questionnaire after signing the informed consent form. A subsample was newly administered the RCTQ-13 5 days after the first administration to evaluate test-retest reliability. To evaluate construct validity through hypothesis testing, a subsample of 55 people was administered the Young Mania Rating Scale (YMRS) [18], the Montgomery–Åsberg Depression Rating Scale (MADRS) [19], the Ruminative Response Scale (RRS) [20], the Penn State Worry Questionnaire (PSWQ) [21], and the State-Trait Anxiety Inventory (STAI) [22]. Discriminant validity was initially approached by comparing the RCTQ-13 scores of the following relevant patient groups: [1] with hypomanic episodes, [2] with manic episodes, 3) with manic episodes with mixed features [4] with depressive episodes, [5] with depressive episodes with mixed features, and [6] euthymic patients (in full remission). However, the hypomanic episode group was not included in the final analysis due to the small number of individuals (*n* = 3) and there were no patients with mania with the mixed symptoms specifier. Therefore, only the euthymia, mania, depression and depression with mixed symptoms groups were left in the final analysis. Classification into each of the groups was determined by an interview conducted by an experienced psychiatrist, using DSM-5 criteria and the results of the Young Mania Rating Scale and the Montgomery–Åsberg Depression Rating Scale. For determining responsiveness, we used a criterion-based approach using the Clinical Global Impression (CGI) rating scale as the reference standard [15]. The RCTQ-13 was administered a second time on a sample of 72 patients 4 weeks after the first administration with CGI for determining change.

#### Instruments

*Short version of the Racing and Crowded Thoughts Questionnaire (RCTQ-13):* 13-item self-report questionnaire that evaluates thought overactivity during the past 24 h [10]. The first 4 items belong to the thought overactivation subscale. The following 4 items belong to the burden of thought overactivation subscale, and the last 5 items correspond to the thought overexcitability subscale.

*Young Mania Rating Scale (YMRS):* it consists of 11 items, which are individually scored on a 5-option response scale corresponding to different degrees of severity of the mania. They are explicitly defined for each item [18]. For each item, the response options are rated with 0, 1, 2, 3, or 4 points. However, the five response options for items 5, 6, 8, and 9 are scored with double points. The final total score of the scale is obtained by adding up all the points, indicating the degree of severity of the patient’s manic state from least to most severe. The scale takes about 15-30 minutes to be administered, and the general recommendation is to mark the highest score applicable to the patient for each item. For this study, we used a cutoff score of > 5 points to determine whether a patient presents hypomania or mania. The scale is not validated for Colombia, but it has been validated in Spanish [18].

*Montgomery-Åsberg Depression Rating Scale (MADRS)*: hetero-administered questionnaire consisting of 10 statements for major depressive episode diagnosis, which focuses on cognitive, affective, and somatic aspects. It has been validated in Spanish and for Colombia [19]. Additionally, 7 degrees of severity (0-6) are considered for each item, which associate the even values (0, 2, 4, 6) to statements. The scale allows for intermediate scores between two statements when it is uncertain which statement applies. The total score of the scale is obtained by adding the values selected for each item, with an interval of 0-60 points.

*Ruminative Response Scale (RRS)*: it is a 22-item self-report questionnaire that evaluates two aspects of rumination during the last 7 days, including the past 24 h: “brooding” (5 items), which refers to the tendency for brooding and mood pondering, is related to a negative mood, and is considered to be maladaptive; and “reflection” (5 items), which refers to active efforts to understand one’s negative feelings, and is considered adaptative. The items are classified on a scale from 1 “almost never” to 4 “almost always.” It is validated for Colombia [20].

*Penn State Worry Questionnaire (PSWQ):* it is a measure of anxiety designed to evaluate the general tendency to experience worry [20]. It consists of 16 items to which participants respond according to a 5-point scale, ranging from 1 (“not at all typical of me”) to 5 (“very typical of me”). The possible range of scores is 16-80: 16-39 = low worry, 40-59 = moderate worry, and 60-80 = high worry. The questionnaire is currently validated for Colombia [21].

*State-Trait Anxiety Inventory (STAI):* instrument based on a theoretical model of anxiety as a state and as a trait [22]. State anxiety is a transient emotional condition characterized by consciously perceived subjective feelings of tension and apprehension, as well as by hyperactivity of the autonomic nervous system. Trait anxiety is a relatively stable personality attribute whereby subjects tend to perceive situations as threatening, consequently raising their anxiety level. The time frame of reference for state anxiety is “right now” (20 items) and was the one used in this study. Each subscale is made up of 20 items on a 4-point Likert scale system based on intensity (0 = almost never/not at all; 1 = somewhat/sometimes; 2 = moderately so/often; 3 = very much so/almost always). The total score in each subscale ranges from 0 to 60 points [21]. It is validated in Spanish and for Colombia [22].

*Clinical Global Impressions (CGI) scale:* it refers to the global impression of the patient and therefore requires clinical experience [23]. It is a descriptive scale that provides qualitative information regarding the severity of the condition and the change seen in the patient compared to the baseline state. It is comprised of two subscales that evaluate the severity of the condition and the improvement of the condition due to treatment. The notion of improvement refers to the distance between the patient’s current condition and the condition recorded at the start of the treatment. Both scales consist of a single item, which in this case was answered by a clinician who evaluated the patients at the time the scales were applied. It is validated in Spanish [23].

### Statistical analysis

To describe the sociodemographic and clinical characteristics of the participating subjects, we used frequencies and percentages for qualitative variables, and medians and interquartile ranges for quantitative variables, since they did not present a normal distribution according to the Shapiro–Wilk test. We also determined the frequency of items with missing data and the frequency of use of each response option.

For structural validity, we conducted a confirmatory factor analysis (CFA) of the three-factor model proposed by the authors using the diagonally weighted least square mean and variance estimator (WLSMV) [11]. The following goodness-of-fit statistics were used: RMSEA (Root Mean Square Error of Approximation), CFI (Comparative Fit Index), TLI (Tucker-Lewis Index), and SRMSR (Standardized Root Mean Square Residual). The fit of the model was considered adequate if: RMSEA = 0.06-0.08 and CFI and TLI > 0.95 [24]. We also evaluated internal consistency using Cronbach’s alpha and McDonald’s omega [25] as well as the correlations between each item and the total score. Test-retest reliability was also determined by means of the ICC with a 95% CI. Also, the Bland–Altman plot was used to represent the limits of agreement between the two measurements for the total score and each factor.

Regarding construct validity through hypothesis testing, convergent validity was assessed by calculating the Spearman correlation coefficient of the RCQT-13 scores with item 7 of the YMRS, which assesses language-thought disorders; a moderate positive correlation was expected. For divergent validity, we calculated the Spearman correlation coefficient between RCTQ-13 and the MADRS, the state subscale of the STAI, and the PSWQ, expecting it to be low as they do not specifically include racing thoughts. While the statistical significance of the Spearman coefficients was calculated, the interpretation was primarily based on the strength of the association. Correlations with values greater than 0.6 are considered as “strong,” those falling between 0.30 and 0.60 are considered “moderate”, and any value below 0.30 suggests a low or weak correlation [26].

For discriminant validity, we compared total and subscale scores between the different patient groups using the Kruskal–Wallis test, as data distribution was not normal. Likewise, ordinal epsilon squared (ε^2^) was calculated for comparing 2 or more groups as a nonparametric effect size measure, with values interpreted as small (0.01-0.06), moderate (0.08-0.26), and large (≥0.26) [27]. Post-hoc pairwise comparison were performed after significant effects with Dunn test with multiple comparison adjustment with Bonferroni method. A level of statistical significance was defined as a *p*-value of less than 0.05.

For responsiveness, we calculated the Spearman correlation coefficient between the change classification indicated in the CGI and the mean difference of the scores obtained in the two measurements of each RCTQ-13 subscale. In addition, the AUROC was calculated for the entire scale, using as a reference the presence of change; it was considered adequate if it presented values > 0.7 [17].

Item response theory was used to estimate the difficulty and discrimination for each item by applying a generalized partial credit model (GPCM) [28, 29]. The category characteristic curve (CCC) was also obtained for each item. The fit was evaluated for each item based on the values of the infit and outfit statistics, which were considered acceptable if they were between 0.5 and 1.5 [30].

The statistical analysis was conducted using Stata 15. For factor analysis and evaluation based on item response theory, we used R [31] and R Studio [32] with the *lavaan* [33] and *ltm* [34] packages, respectively.

## Results

### Translation, adaptation and pilot test

We obtained a Spanish RCTQ-13 version which was approved by the review board. It proved to be easy to administer in the pilot test, although supervision was required for a few subjects with a low level of education. Thus, it was decided that participants must have completed until the fifth year of elementary school to participate in the rest of the study. The average time for administering the questionnaire was 5.1 minutes. In the cognitive interview, certain items presented comprehensibility issues, which led to modifications. Item 2 was changed from *“Mis pensamientos van a 200km/h”* (My thoughts race at 200 km/h) to *“Mis pensamientos van muy rápido”(My thoughts go very fast)* because the symbol “km/h” was confusing. Item 5 was also modified from *“Mi cerebro no puede manejar todos los pensamientos que me surgen al mismo tiempo”* (My brain cannot manage all these thoughts that arise at the same time) to *“Mi cerebro no puede controlar todos los pensamientos cuando me surgen al mismo tiempo”* (My brain cannot control all thoughts when they come to me at the same time) because the participants had difficulty understanding the word “manage” specifically associated with mental capacity. Item 6, *“Me siento angustiado en mi vida diaria por la gran cantidad de pensamientos o por la velocidad de estos en mi mente”* (I feel distressed in my everyday life by the great number of thoughts or by the velocity of thoughts in my mind) was changed to *“Me angustia tener tantos pensamientos en la mente y/o que vayan tan rápido” (I feel distressed by so many thoughts in my mind and/or to have them go so fast)* because the participants found the item to be too long and complex. These modifications were evaluated in a new group of 10 patients. They found it easy to understand, and the version was submitted for validation (The complete scale in Spanish is available in Additional file 1).

### Validation process

A total of 250 participants were included, 22% of whom were male, with a median age of 37.5 years and an 11-year level of education. In the clinical interview, 190 patients were diagnosed with BD type I (76%), mainly in a manic episode. Other demographic and clinical characteristics are shown in Table 1. There were no unanswered items, and the participants used all the response options in each item (Table 2).

**Table 1:** Sociodemographic and clinical characteristics of the participants (*n* = 250)

**Characteristic**	**Frequency**	**Percentage**
Male	55	22
Urban housing	198	79.2
Occupation		
*Unemployed*	57	22.8
*Employed*	79	31.6
*Homemaker*	73	29.2
*Pensioner*	41	16.4
Unmarried	163	65.2
Diagnosis		
*Bipolar I Disorder*	190	76
*In full remission (euthymic)*	25	10
*Current episode manic*	123	49.2
*Current episode hypomanic*	3	1.2
*Current episode depressed*	14	5.6
*Current episode depressed with mixed features*	25	10
* Major depressive disorder*	60	24.0
*Current depressed episode*	59	23.6
*Current depressed episode with mixed features*	1	0.4
Comorbidity^a^		
*Substance use disorder*	57	23.8
*Anxiety disorder*	20	8.0
*Personality disorder*	12	4.8
*Other*	6	2.4
Medications^a^		
*Stabilizers*	180	72.0
*Antipsychotics*	195	78.3
*Antidepressants*	59	23.7
	**Median**	**Interquartile range**
Age	37.5	27.0-56.0
Level of education	11	6.0-11.0
Young Mania Rating Scale	25	0.0-36.0
Ruminative Response Scale	53	34.0-66.0
Penn State Worry Questionnaire	35	24.0-46.0
State-Trait Anxiety Inventory	29	23.0-32.0

^a^ Not mutually exclusive

**Table 2:** Frequency of responses to each item of the Spanish version of the RCTQ-13 (*n* = 250)

Item	Response options				
	Not at all	Somewhat agree	Moderately agree	Agree	Completely agree
	Frequency (%)	Frequency (%)	Frequency (%)	Frequency (%)	Frequency (%)
1	39 (15.6)	28 (11.2)	31 (12.4)	58 (23.3)	94 (37.8)
2	46 (18.0)	21 (8.4)	36 (14.5)	64 (25.8)	83 (33.3)
3	53 (21.2)	21 (8.4)	30 (12.0)	68 (27.3)	78 (31.3)
4	53 (21.2)	21 (8.4)	24 (9.7)	61 (24.5)	91 (36.5)
5	92 (36.8)	32 (13.0)	31 (12.4)	38 (15.2)	57 (23.0)
6	106 (42.4)	20 (8.0)	24 (9.6)	30 (12.0)	70 (28.0)
7	110 (44.0)	17 (6.8)	30 (12.0)	37 (14.8)	56 (22.4)
8	68 (27.2)	33 (13.2)	32 (12.8)	41 (16.4)	76 (30.4)
9	71 (28.4)	45 (18.0)	30 (12.0)	52 (20.8)	52 (20.8)
10	99 (39.6)	34 (13.6)	32 (12.8)	37 (14.8)	48 (19.2)
11	57 (22.8)	32 (12.8)	39 (15.6)	58 (23.2)	64 (25.6)
12	84 (33.6)	26 (10.4)	40 (16.0)	42 (16.8)	58 (23.2)
13	90 (36.0)	20 (8.0)	36 (14.4)	29 (11.6)	75 (30.0)

### Structural validity

**Fig. 1:**
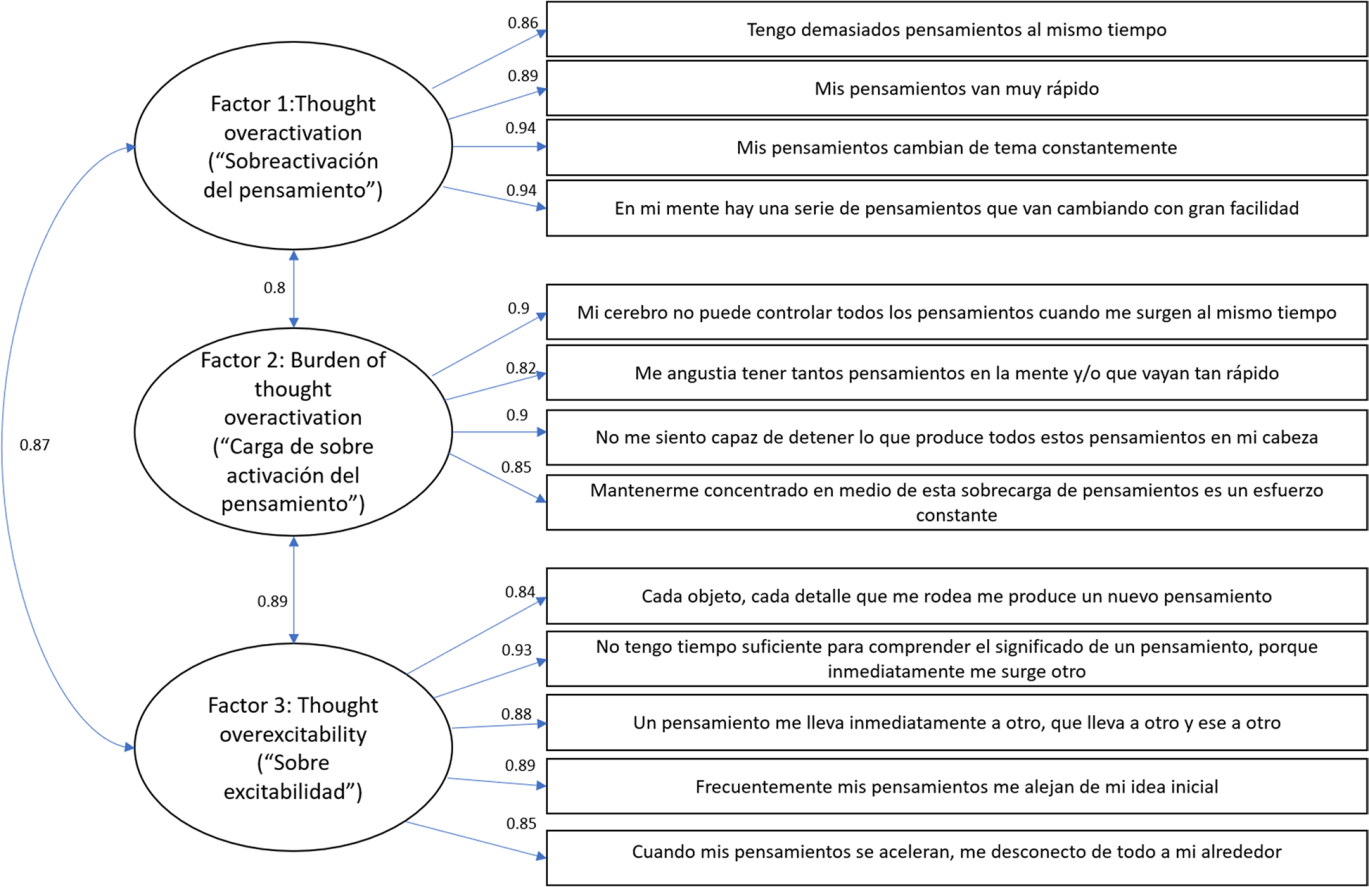
Confirmatory factor analysis of the Spanish version of the RCTQ-13. Three-factor structure displaying correlations between items and factors, for which the goodness of fit was good (Root Mean Square Error of Approximation = 0.061, Comparative Fit Index = 0.9, Tucker-Lewis = 0.9, and Standardized Root Mean Square Residual = 0.04)

The three-factor structure hypothesis proposed by the developers of the scale was confirmed in the CFA (Fig. 1), with goodness-of-fit statistics that were adequate for the model (RMSEA = 0.061, CFI = 0.9, TLI = 0.9, and SRMSR = 0.04).

### Reliability

Internal consistency was adequate for the entire scale and for each of the factors (Table 3).

**Table 3:** Internal consistency of the Spanish version of the RCTQ-13 evaluated in a Colombian population

Scale component		Item Correlation- Total	Cronbach’s alpha (95% IC)	McDonald’s omega (95% IC)
Factor #1. Thought overactivation	Item 1	0.73	0.92 (0.89 – 0.94)	0.95 (0.89 – 0.94)
	Item 2	0.76		
	Item 3	0.77		
	Item 4	0.78		
Factor #2. Burden of thought overactivation	Item 5	0.76	0.88 (0.85 - 0.91)	0.89 (0.84 - 0.90)
	Item 6	0.69		
	Item 7	0.73		
	Item 8	0.73		
Factor #3. Overexcitability	Item 9	0.73	0.88 (0.88 – 0.92)	0.92 (0.88 – 0.94)
	Item 10	0.81		
	Item 11	0.78		
	Item 12	0.77		
	Item 13	0.73		
Total Scale			0.95 (0.94 - 0.96)	0.95 (0.94 - 0.96)

**Fig. 2:**
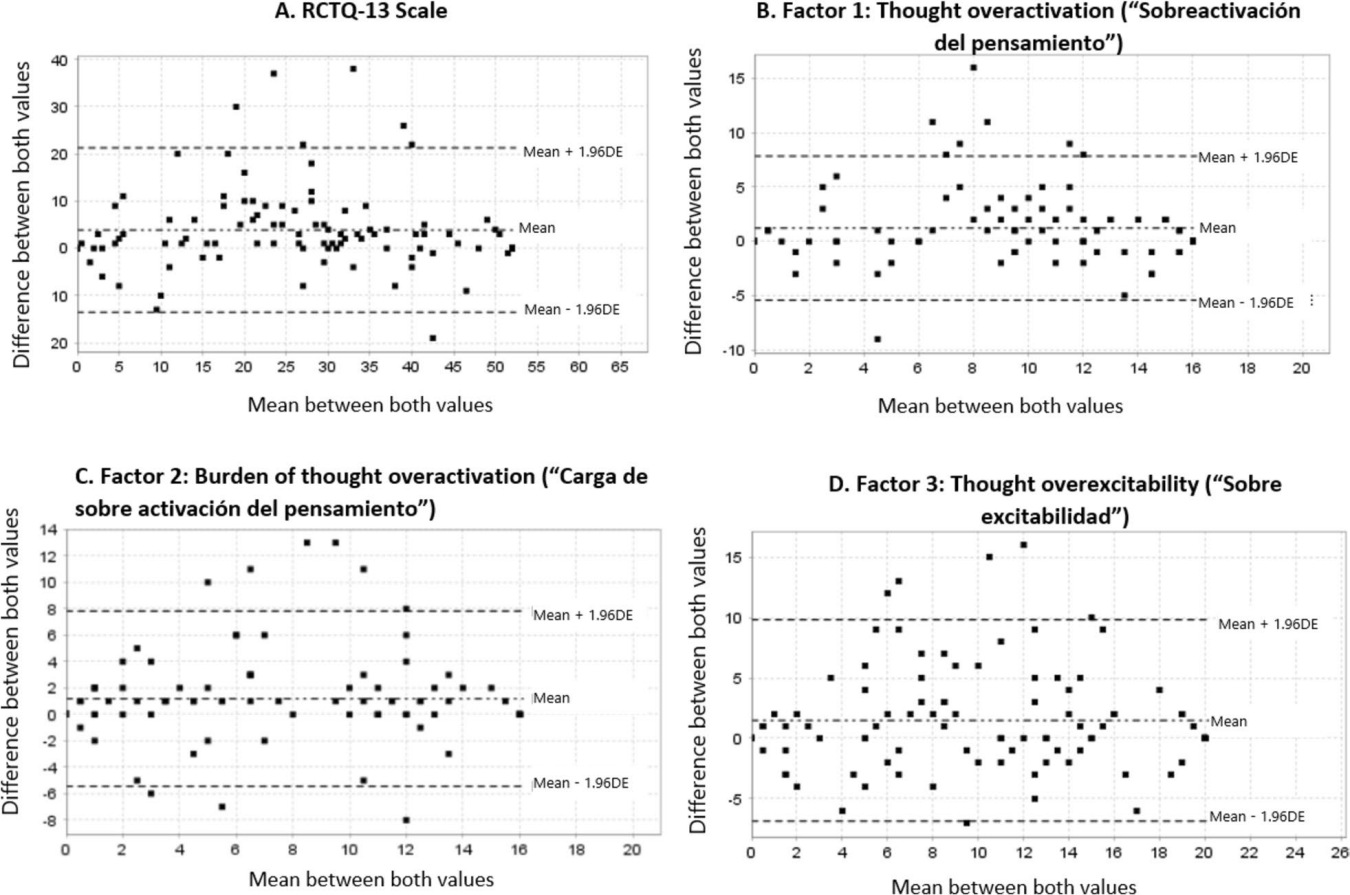
Bland–Altman plots for test-retest reliability of the Spanish version of the RCTQ-13 and its factors. **A** Entire scale (Racing and Crowded Thoughts Questionnaire 13 items). Mean difference: 3.8 (95% CI: 2.1 to 5.6). Limits of agreement: -13.5 (95% CI: − 16.5 to − 10.5) and 21.2 (95% CI: 18.3 to 24.2). **B** Factor #1. Mean difference: 1.2 (95% CI: 0.5 to 1.8). Limits of agreement: -5.4 (95% CI: − 6.5 to − 4.2) and 7.8 (95% CI: 6.7 to 8.9). **C** Factor #2. Mean difference: 1.1 (95% CI: 0.5 to 1.8). Limits of agreement: -5.4 (95% CI: − 6.5 to − 4.3) and 7.8 (95% CI: 6.6 to 8.9). **D** Factor #3. Mean difference: 1.4 (95% CI: 0.6 to 2.3). Limits of agreement: -6.8 (95% CI: − 8.2 to − 5.4) and 8.8 (95% CI: 8.3 to 11.2)

Regarding test-retest reliability, the ICC for the entire RCTQ-13 was 0.82 CI (95% CI 0.70-0.88); 0.79 (95% CI 0.68-0.86) for Factor #1, 0.80 (95% CI 0.70-0.87) for Factor #2, and 0.77 (95% CI 0.66-0.85) for Factor #3. The Bland–Altman plot for the entire scale and each of the factors showed that there are slight differences between the two administrations with a slightly higher score in the first evaluation, especially in the middle range of scores, with no observable systematic trend (Fig. 2).

### Construct validity

According to the convergent validity, there was a low negative correlation of the RCTQ-13 scale with the scores of item 7 of the YMRS (Table 4). Regarding divergent validity, the total scale and the three factors showed a low positive correlation with the MADRS and the STAI, as was expected. However, there were moderate positive correlations with the PSWQ and the RRS (Table 4).

**Table 4:** Convergent and divergent construct validity of the Spanish version of the RCTQ-13 in a Colombian population

Score	RCTQ total	Factor #1	Factor #2	Factor #3
Young Mania Rating Scale	−0.10	− 0.05	− 0.17	− 0.08
Young Mania Rating Scale, Item 7	**−0.12** ^*^	− 0.01	**−0.18** ^*^	− 0.06
Montgomery–Åsberg Depression Rating Scale	**0.23** ^**^	−0.08	**0.29** ^**^	**0.19** ^*^
Ruminative Response Scale	**0.42** ^**^	0.11	**0.47** ^**^	**0.38** ^**^
Penn State Worry Questionnaire	**0.55** ^**^	**0.38** ^**^	**0.60** ^**^	**0.48** ^**^
State-Trait Anxiety Inventory	0.09	0.01	0.20	0.05

The value represents Spearman’s rho coefficient

* *p* < 0,05

** *p* < 0,001

**Fig. 3:**
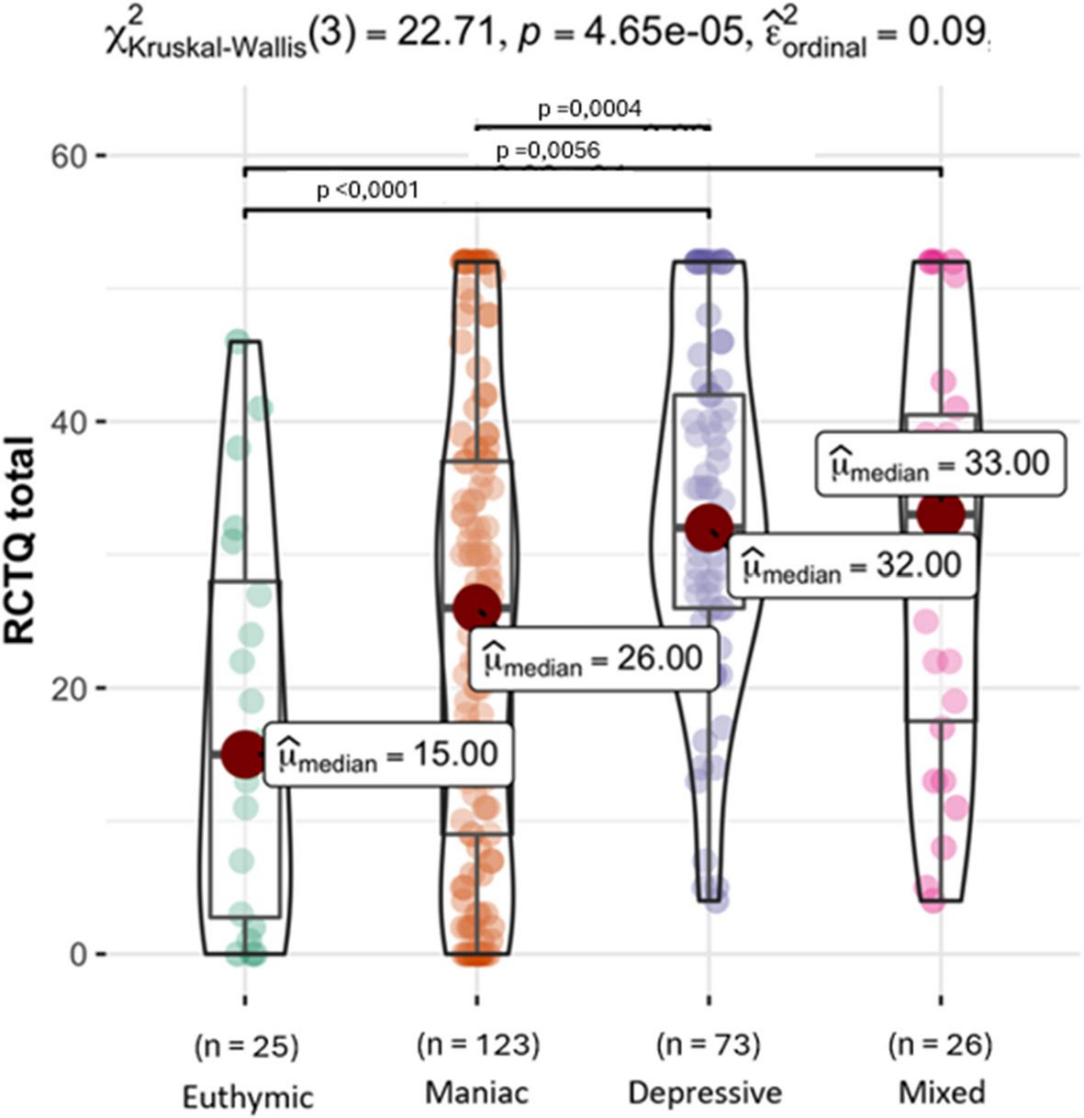
Discriminant validity of the RCQT-13. Median total scores of the patients with different affective episodes (*n* = 247). Medians were compared using Kruskal-Wallis test, which showed statistically significant differences, with moderate effect size so post-hoc paired comparisons were performed using Dunn’s test, applying a Bonferroni correction for multiple comparisons. The statistically significant pairwise comparisons are shown at the top. Patients with current episode hypomanic (*n* = 3) were not included

As for discriminant validity, statistically significant differences were found in the total score of the scale and between patients with different affective episodes, with a moderate effect size (ε^2^ = 0.09) (Fig. 3).

Individuals in the groups experiencing depressive and depressive with mixed features episodes had higher total scale scores compared to those in the groups with manic episodes and euthymic states (Table 5). Specifically, the groups with depressive with and without mixed features episodes had higher scores in relation to Factor #2 (Burden of thought overactivation) when compared to individuals in the manic episode and euthymic groups, suggesting that the difference in total score is at the expense of this factor. As expected, the euthymia group scored lower on the total scale and on all factors.

**Table 5:** Discriminant construct validity of the Spanish version of the RCTQ-13 in a Colombian population with different affective episodes (*n* = 247)^a^

Score	1 Euthymia (***n*** = 25)	2 Maniac (***n*** = 123)	3 Depressive (***n*** = 73)	4 Mixed features (***n*** = 26)	Kruskal-Wallis test ***p***-value	Dunn’s Pairwise Comparison p-value (Bonferroni correction)					
	Median (IQR)	Median (IQR)	Median (IQR)	Median (IQR)		1 vs 2	1 vs 3	1 vs 4	2 vs 3	2 vs 4	3 vs 4
RCTQ total	15 (2.5 – 29.0)	26 (9.0 – 37.0)	32 (26.0 – 42.0)	33 (17.0 – 41.0)	**0.0001**	0.0708	**< 0.0001**	**0.0056**	**0.0004**	0.2452	1.0000
Factor #1	7 (1.5 – 11.5)	11 (4.0 – 15.0)	12 (9.0 – 16-0)	11.5 (10.0 –15.0)	**0.0029**	**0.0057**	**0.0001**	**0.0035**	0.1365	0.9026	1.0000
Factor #2	3 (0.0 – 9.0)	4 (1.0 – 11.0)	11 (7.0 – 14.0)	11.5 (2.0 – 14.0)	**0.0001**	0.3721	**< 0.0001**	**0.0250**	**< 0.0001**	0.1806	0.3517
Factor #3	6 (1.0 – 10.0)	10 (1.0 – 15.0)	11 (7.0 – 16.0)	11 (7.0 – 15.0)	**0.0016**	0.1396	**0.0005**	0.0114	0.2444	0.2444	1.0000

^a^ Patients with current episode hypomanic (*n* = 3) were not included

*IQR* interquartile range

### Responsiveness

The correlation between the change in the CGI and the mean difference in RCTQ-13 scores was moderate and negative (ICC = − 0.31). Based on the total scores of the RCTQ-13 scale and the outcome of change or no change, according to the CGI, the area under the ROC curve was 0.71 (95% CI 0.50-0.92).

### Item response theory

**Fig. 4:**
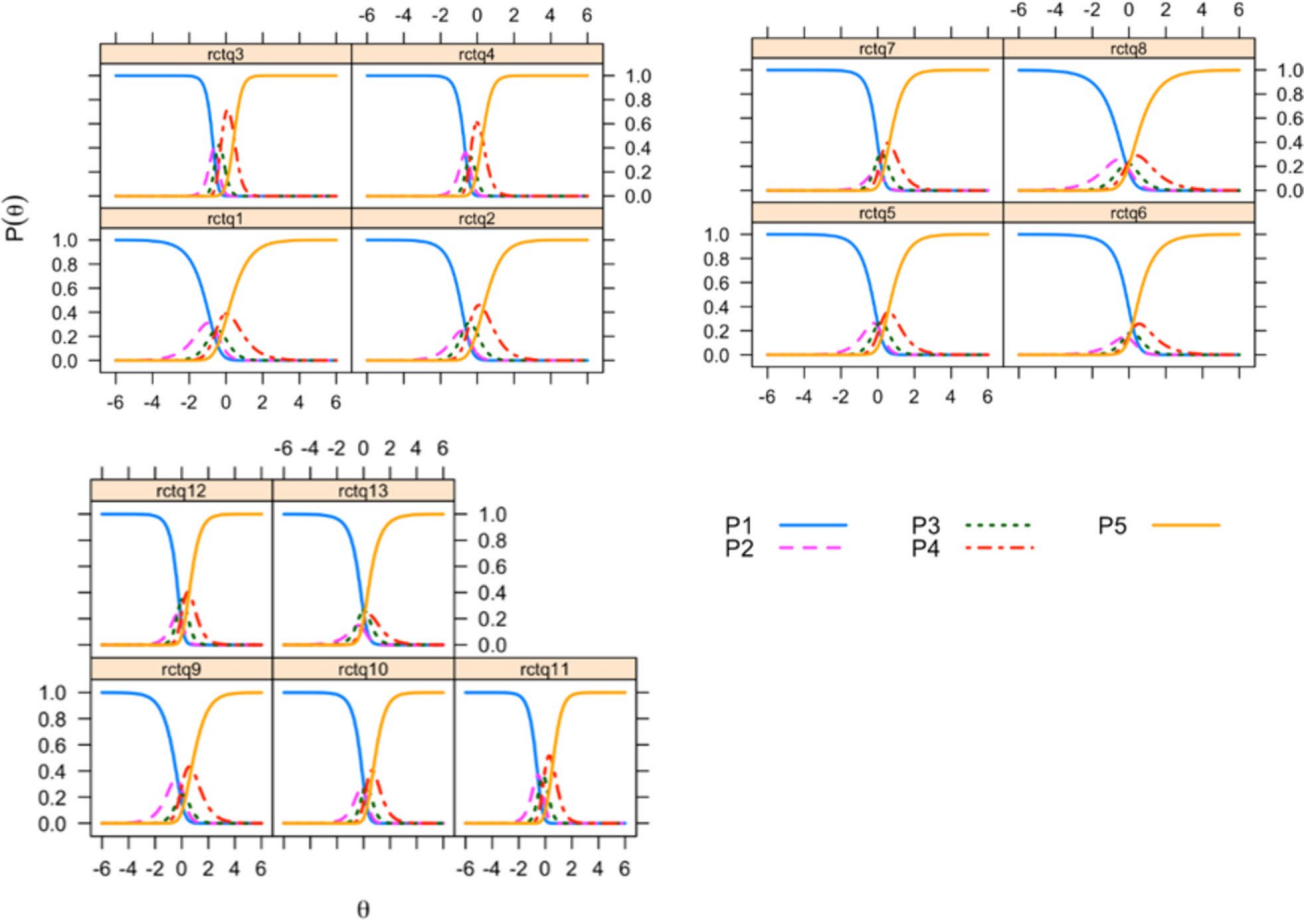
Category characteristic curve for each item of the Spanish version of the RCTQ-13. The number for each item corresponds to the number after “rctq”. Item 10, for example, is rctq10. Each option for the response of the item es presented as a specific curve, and is designated with the letter P. First response option “Not at all”, for example, is P1

Upon analyzing item difficulty, we found that item 1 *“Tengo demasiados pensamientos al mismo tiempo”* (I have too many thoughts at the same time) was the easiest, while item 10 *“No tengo tiempo suficiente para comprender el significado de un pensamiento, porque inmediatamente me surge otro”* (There is not enough time to grasp the meaning of a thought, as new ones immediately arise) was the most difficult. The CCCs for each item are presented in Fig. 4. In general, the response thresholds for the response options are organized.

The infit was adequate for all items and the outfit was acceptable, except for items 3, 4, and 7 (Table 6).

**Table 6:** Item response theory parameters of the Spanish version of the RCQT-13 in a Colombian population (*n* = 250)

RCQT-13		Difficulty	Infit	Outfit
Factor #1	Item 1	−0.45	0.94	0.78
	Item 2	−0.34	0.94	0.72
	Item 3	−0.26	0.70	0.43
	Item 4	−0.31	0.68	0.43
Factor #2	Item 5	0.19	0.81	0.59
	Item 6	0.18	0.85	0.63
	Item 7	0.25	0.69	0.43
	Item 8	−0.07	0.88	0.70
Factor #3	Item 9	0.09	0.90	0.80
	Item 10	0.26	0.86	0.62
	Item 11	−0.11	0.78	0.57
	Item 12	−0.09	0.84	0.58
	Item 13	−0.04	0.93	0.69

## Discussion

We linguistically and culturally adapted the short version of the RCTQ-13, and we found that it has a structure coherent with the theoretical development of the instrument, adequate internal consistency, and test-retest reliability in patients with affective disorders. Evidence of its discriminant construct validity was also observed, as the hypothesis of differences between euthymic patients and those with affective episodes was met. In our study, most participants were experiencing a manic episode. This differs from the original RCTQ-13 validation study, which did not include patients with mania and had a larger sample of patients with hypomania [10]. Despite this, we found evidence that the Spanish version of RCTQ-13 measure the construct intended to be measured, as it captures not only racing thoughts but also crowded thoughts.

The concept of detecting racing thought only in episodes of mania and hypomania has been expanded with the evidence that in depression there is also the psychopathological description of racing thoughts. This symptom would not only be a specifier or an indication of bipolar depression. In patients with unipolar depression, it has been seen that up to 56.4% may experience racing/crowded thinking [5]. For some clinicians, the often-unrecognized description of these thought symptom can even guide the pharmacological treatment of unipolar depression [35]. Precisely in our study, patients with depressive disorder scored higher on the RCTQ-13, even without having the specifier of mixed symptoms. This would be in favor of racing and crowded thoughts being expressed in all affective disorders as a broad spectrum [36, 37], but that could be expressed with different nuances.

In this regard, the depressive episodes groups had statistically higher scores than the other groups on Factor #2 (“Burden of thought overactivation”). And when evaluating the divergent construct validity of RCTQ-13, a moderate positive correlation was found with the PSWQ AND RRS, especially with this Factor #2. Thus, as previous studies have suggested, racing thoughts in depression could generate great emotional distress and are related to rumination [38–40], that is perceived as a “crowded” type thinking that the patient often describes as their head being full of thoughts they cannot stop [38]. To that extent, it is possible that the experience itself may constitute a specific focus of concern for the subject. And therefore, it is necessary to maintain the distinction crowded thought as a specific subtype of racing thoughts and to continue studying relationship between the racing thoughts construct with rumination and worry.

An important finding in this study is that patients with a manic episode scored higher than patients in euthymia specifically on Factor #1, which has to do with thought overactivation, and would indicate a specific facet of racing thoughts in mania. With all this, we think that the idea that racing thoughts constitute a multifaceted phenomenon existing differentially along a continuum across the spectrum of symptomatic mood states is supported [41]. As we evidenced that the patients with mania had statistically lower total scores than patients with depression and depression with mixed symptoms, it is possible that the experience of racing thoughts is different in this group. We observed that the correlation between the Spanish version of the RCTQ-13 and the racing thoughts assessed by clinicians in item 7 of the YMRS, was small and negative. We believe that this difference has to do with the patients’ subjective experience of racing thoughts, which could be different from what the clinician perceives when scoring the YMRS. It is possible that, for patients in a manic state, who were the majority, this experience of acceleration could be pleasant or normalized and their scores on the scale do not reflect the degree of disturbance perceived by the clinician and without the burden (Factor #2) of depressive episodes. It is also possible that the patient may have a perception of increase of thought velocity, but that the clinician did not perceive it in their language assessment.

This discrepancy in the assessment of thought experience between the patient and the clinician has also been suggested by Goldberg [42], who found very low concordance (coefficient κ = 0.15) on the racing thought item of the Mood Disorder Questionnaire (MDQ). This highlights the complexity of approaching thought assessment, where an interaction occurs between the patient’s perception as internal subjective experience and the external detection that the clinician can make in the clinical evaluation. Another possible explanation is that some patients may have been under the effects of sedatives, given that enrollment occurred during inpatient care of the acute episode. Therefore, despite experiencing acceleration, the clinician may not have been able to perceive it due to drowsiness or dysarthria. We do not consider that we have found an indication of a lack of construct validity, but rather that there is a research opportunity to delve deeper into racing thoughts as a subjective experience and how it is reflected in clinical examination.

We also found that the RCTQ-13 has adequate responsiveness when applied a month after the first assessment. This finding is noteworthy, since the authors did not analyze this psychometric property in patients with mood disorders [8, 10]. Responsiveness holds particular significance in longitudinal patient follow-ups as it denotes the capacity of a measurement instrument to detect changes over time in the targeted construct [43], and it is a fundamental psychometric property so that measurement instruments can be used to measure outcomes in clinical trials [44]. In this sense, we have provided evidence of the responsiveness of the RCTQ-13 and its use could be possible to measure changes in patients before and after treatment or for follow-up over time.

We also conducted an analysis of the Spanish version of the RCTQ-13 using IRT, which had not been done before. One of the main advantages of this approach is that it allows knowing the difficulty of the items and the trait level in the measured individuals and it has become an important and complementary approach in the validation process of scales that measure psychological constructs [45]. One of its advantages is that it helps determine how much of the racing thought experience is required to answer each item. With this information, it is possible to select items for different purposes and populations. If, for example, a clinician wanted to screen for experience in the general population, where the amount of the trait is expected to be low, they could use the easiest items, such as item 1 and item 2, which generically inquire on thought overactivation. However, for the assessment of severity and classification of patients with affective episodes, more difficult items should be used, such as item 10, which requires much more of the trait to provide an answer. It is important to note that items 3, 4, and 7 had a low outfit. This could indicate that these items do not fit the model well and do not represent the outliers. This may be due to the fact that the participants made careless mistakes or guessed, which is to be expected to an extent in manic episodes (the most frequent in our study) and may also have contributed to the lower total score obtained in our study. We could therefore suggest that, for this subgroup of patients, the supervision of the clinician could be required or that the possible elimination of these items should be reviewed.

An important limitation in our study was the low representation of patients experiencing a hypomanic episode, which makes it difficult to directly compare our adaptation with the developmental studies of the original version of the scale. It also does not allow us to establish differences in the subjective experience of racing thoughts between patients experiencing these episodes and in other affective states.

## Conclusion

The Spanish version of the RCTQ-13 adapted for the Colombian population has adequate reliability and construct validity and responsiveness. Thus, it can be used to measure the construct of racing and crowded thoughts in patients with the spectrum of affective disorders in whom this experience can be expressed with different nuances. It is important to continue studying the racing thoughts construct, considering its relationship with rumination and worry.

### Supplementary Information

**Supplementary material 1.**

## Data Availability

The datasets generated for this study are available on request to the corresponding author.

## Abbreviations

BD: Bipolar disorder
CCC: Category characteristic curve
CFI: Comparative fit index
GPCM: Generalized partial credit model
ICC: Intraclass correlation coefficient
IRT: Item response theory
MADRS: Montgomery-Åsberg depression rating scale
PSWQ: Penn State worry questionnaire
RCTQ-13: Racing and crowded thoughts questionnaire, 13-items version
RMSEA: Root mean square error of approximation
RRS: Ruminative response scale
SRMSR: Standardized root mean square residual
STAI: State-Trait anxiety inventory
TLI: Tucker-Lewis index
YMRS: Young Mania rating scale

## References

[1] Piguet C, Dayer A, Kosel M, Desseilles M, Vuilleumier P, Bertschy G (2010). Phenomenology of racing and crowded thoughts in mood disorders: a theoretical reappraisal. J Affect Disord.

[2] Goodwin FK, Jamison KR (2007). Manic-Depressive Illness: Bipolar Disorders and Recurrent Depression. Oxford University Press, editor. USA.

[3] Braden W, Ho CK (1981). Racing thoughts in psychiatric inpatients. Arch Gen Psychiatry.

[4] Benazzi F (2003). Depression with racing thoughts. Psychiatry Res.

[5] Benazzi F (2005). Unipolar depression with racing thoughts: a bipolar spectrum disorder?. Psychiatry Clin Neurosci.

[6] Martz E, Bertschy G, Kraemer C, Weibel S, Weiner L (2021). Beyond motor hyperactivity: racing thoughts are an integral symptom of adult attention deficit hyperactivity disorder. Psychiatry Res.

[7] Bertschy G, Weibel S, Giersch A, Weiner L (2020). Racing and crowded thoughts in mood disorders: a data-oriented theoretical reappraisal. Encephale..

[8] Weiner L, Weibel S, de Sousa GW, Keizer I, Gex-Fabry M, Giersch A (2018). Measuring racing thoughts in healthy individuals: the racing and crowded thoughts questionnaire (RCTQ). Compr Psychiatry.

[9] de Dios C, Goikolea JM, Colom F, Moreno C, Vieta E (2014). Los trastornos bipolares en las nuevas clasificaciones: DSM-5 y CIE-11. Rev Psiquiatr Salud Ment.

[10] Weiner L, Ossola P, Baptiste CJ, Desseilles M, Keizer I, Yves MJ (2019). Racing thoughts revisited: a key dimension of activation in bipolar disorder. J Affect Disord.

[11] Weiner L, Martz E, Kilic-Huck Ü, Siegel N, Bertschy G, Geoffroy PA, et al. Investigating racing thoughts in insomnia: a neglected piece of the mood-sleep puzzle? Compr Psychiatry. 2021;111. 10.1016/j.comppsych.2021.152271 34555554

[12] Patrick DL, Burke LB, Gwaltney CJ, Leidy NK, Martin ML, Molsen E (2011). Content validity — establishing and reporting the evidence in newly developed patient-reported outcomes (PRO) instruments for medical product evaluation: ISPOR PRO good research practices task force report: part 2 — assessing respondent understanding. JVAL.

[13] American Psychiatry Association. Diagnostic and statistical manual of mental disorders. In: DSM-5. Editorial Médica Panamericana. Fifth ed. Madrid; 2013.

[14] Streiner DL, Norman GR (2015). Health measurement scales.

[15] de Vet HCW, Terwee CB, Mokkink LB, Knol DL (2011). Measurement in medicine a practical guide.

[16] Machin D, Campbell MJ, Fayers P, Pinol A (1997). Sample size tables for clinical studies.

[17] Hanley JAMB (1982). The meaning and use of the area under a receiver operating characteristic (ROC) curve. Radiology..

[18] Colom F, Vieta E, Martínez-arán A (2002). Versión española de una escala de evaluación de la manía: validez y fiabilidad de la Escala de Young the Young Mania Rating Scale. Med Clin (Barc) [Internet].

[19] Cano JF, Gomez C, Rondón M (2015). Validación en Colombia del instrumento para evaluación de la depresión Montgomery - Åsberg Depression Rating Scale (MADRS). Rev Colomb Psiquiatr.

[20] Ruiz F, Suárez JC, Sierra MA, Barreto KD, Garcia MB, Bernal PA, Ramírez ES (2017). Propiedades psicométricas y la estructura factorial de la Escala-Formulario Corto Respuestas Ruminativas en Colombia. Int J Psychol Psychol Ther.

[21] Ruiz FJ, Monroy-Cifuentes A, Suárez-falcón JC (2018). Penn State worry Questionnaire-11 validity in Colombia and factorial equivalence across gender and nonclinical and clinical samples. Anal Psicol.

[22] Castrillón DA, Borrero PE (2005). Validación del inventario de ansiedad estado-rasgo (STAIC) en niños escolarizados entre los 8 y 15 años. Acta colombiana de psicología.

[23] Casas E, Escandell MJ, Ribas M, Ochoa S (2010). Instrumentos de evaluación en rehabilitación psicosocial. Rev Asoc Esp Neuropsiq.

[24] Hu L, Bentler PM (1999). Cutoff criteria for fit indexes in covariance structure analysis: conventional criteria versus new alternatives. Struct Equ Model.

[25] Trizano-Hermosilla I, Alvarado JM (2016). Best alternatives to Cronbach's alpha reliability in realistic conditions: congeneric and asymmetrical measurements. Front Psychol.

[26] Akoglu H (2018). User's guide to correlation coefficients. Turk J Emerg Med.

[27] R Handbook: Kruskal–Wallis Test. 18 Jan. 2022 Available from: rcompanion.org/handbook/F_08.html.

[28] Desjardins CD, Bulut O (2018). Handbook of educational measurement and psychometrics using R.

[29] Paek I, Cole K (2019). Using R for item response theory model applications.

[30] Linacre JM (2017). Teaching Rasch measurement. Rasch Measurement Transactions.

[31] R Core Team (2022). R: a language and environment for statistical computing.

[32] Studio Team (2020). RStudio: integrated development for R.

[33] Rosseel Y (2012). Lavaan: an R package for structural equation modeling. J Stat Softw.

[34] Rizopoulos D (2006). Ltm: an R package for latent variable modelling and item response theory analyses. J Stat Softw.

[35] Wilf TJ (2019). Racing thoughts: what to consider. Curr Psychiatr Ther.

[36] Cassano GB, Rucci P, Frank E, Fagiolini A, Dell’Osso L, Shear MK (2004). The mood spectrum in unipolar and bipolar disorder: arguments for a unitary approach. Am J Psychiatry.

[37] Corponi F, Anmella G, Pacchiarotti I, Samalin L, Verdolini N, Popovic D, Azorin JM, Angst J, Bowden CL, Mosolov S, Young AH, Perugi G, Vieta E, Murru A (2020). Deconstructing major depressive episodes across unipolar and bipolar depression by severity and duration: a cross-diagnostic cluster analysis on a large, international, observational study. Transl Psychiatry.

[38] Koukopoulos A (1999). Agitated depression as a mixed state and the. Psychiatr Clin North Am.

[39] Benazzi F, Akiskal HS (2006). Psychometric delineation of the most discriminant symptoms of depressive mixed states. Psychiatry Res.

[40] Balázs J, Benazzi F, Rihmer Z, Rihmer A, Akiskal KK, Akiskal HS (2006). The close link between suicide attempts and mixed (bipolar) depression: implications for suicide prevention. J Affect Disord.

[41] Bertschy G, Martz E, Weibel S, Weiner L (2023). Psychopathological dissection of bipolar disorder and ADHD: Focussing on racing thoughts and verbal fluency. Neuropsychiatr Dis Treat.

[42] Goldberg JF, Garakani A, Ackermann SH (2012). Clinician-rated versus self-rated screening for bipolar disorder among inpatients with mood symptoms and substance misuse. The Journal of Clinical Psychiatry.

[43] Mokkink L, Terwee C, de Vet H (2021). Key concepts in clinical epidemiology: responsiveness, the longitudinal aspect of validity. J Clin Epidemiol.

[44] Revicki DA, Cella D, Hays RD, Sloan JA, Lenderking WR, Aaronson NK. Responsiveness and minimal important differences for patient reported outcomes. Health Qual Life Outcomes. 2006;4(70) 10.1186/1477-7525-4-70. 10.1186/1477-7525-4-70 PMC1586195 17005038

[45] Cai L, Choi K, Hansen M, Harrell L (2016). Item response theory. Annual Review of Statistics and Its Application.

