# Supplementary material for: Spanish validation of the short version of the racing and crowded thoughts questionnaire (RCTQ-13)
Source: BMC Psychiatry. 2024 Mar 19;24:214. doi: 10.1186/s12888-024-05618-1 (PMC10953190; doi:10.1186/s12888-024-05618-1)
Supplement: Supplementary file 1 — Supplementary material 1. [file 12888_2024_5618_MOESM1_ESM.doc]

**Additional File 1: Spanish version of the Racing and Crowded Thoughts Questionnaire**

**Cuestionario de pensamientos acelerados y amontonados**

**En este cuestionario se describen experiencias que suceden frecuentemente en el pensamiento de personas que cursan con algún trastorno del ánimo. Estas experiencias tienen que ver con la velocidad con la que se mueven sus pensamientos y la cantidad de pensamientos que ha tenido en las últimas 24 horas. Para llenar el cuestionario, usted deberá marcar con una X en la casilla que usted considere que se acomoda más al grado de acuerdo que usted tiene con la frase que describe lo que usted ha estado experimentando durante el último día.**

|  | **Ítem** | **No estoy de acuerdo** | **Algo de acuerdo** | **Moderadamente de acuerdo** | **De acuerdo** | **Completamente de acuerdo** |
| --- | --- | --- | --- | --- | --- | --- |
| 1 | Tengo demasiados pensamientos al mismo tiempo |  |  |  |  |  |
| 2 | Mis pensamientos van muy rápido |  |  |  |  |  |
| 3 | Mis pensamientos cambian de tema constantemente |  |  |  |  |  |
| 4 | En mi mente hay una serie de pensamientos que van cambiando con gran facilidad |  |  |  |  |  |
| 5 | Mi cerebro no puede controlar todos los pensamientos cuando me surgen al mismo tiempo |  |  |  |  |  |
| 6 | Me angustia tener tantos pensamientos en la mente y/o que vayan tan rápido |  |  |  |  |  |
| 7 | No me siento capaz de detener lo que produce todos estos pensamientos en mi cabeza |  |  |  |  |  |
| 8 | Mantenerme concentrado en medio de esta sobrecarga de pensamientos es un esfuerzo constante |  |  |  |  |  |
| 9 | Cada objeto, cada detalle que me rodea me produce un nuevo pensamiento |  |  |  |  |  |
| 10 | No tengo tiempo suficiente para comprender el significado de un pensamiento, porque inmediatamente me surge otro |  |  |  |  |  |
| 11 | Un pensamiento me lleva inmediatamente a otro, que lleva a otro y ese a otro |  |  |  |  |  |
| 12 | Frecuentemente mis pensamientos me alejan mucho de mi idea inicial |  |  |  |  |  |
| 13 | Cuando mis pensamientos se aceleran, me desconecto de todo a mi alrededor |  |  |  |  |  |
